# Supplementary figures and images for: Oxydifficidin, a potent Neisseria gonorrhoeae antibiotic due to DedA-assisted uptake and ribosomal protein RplL sensitivity
Source: eLife. 2025 May 28;13:RP99281. doi: 10.7554/eLife.99281 (PMC12119084; doi:10.7554/eLife.99281)

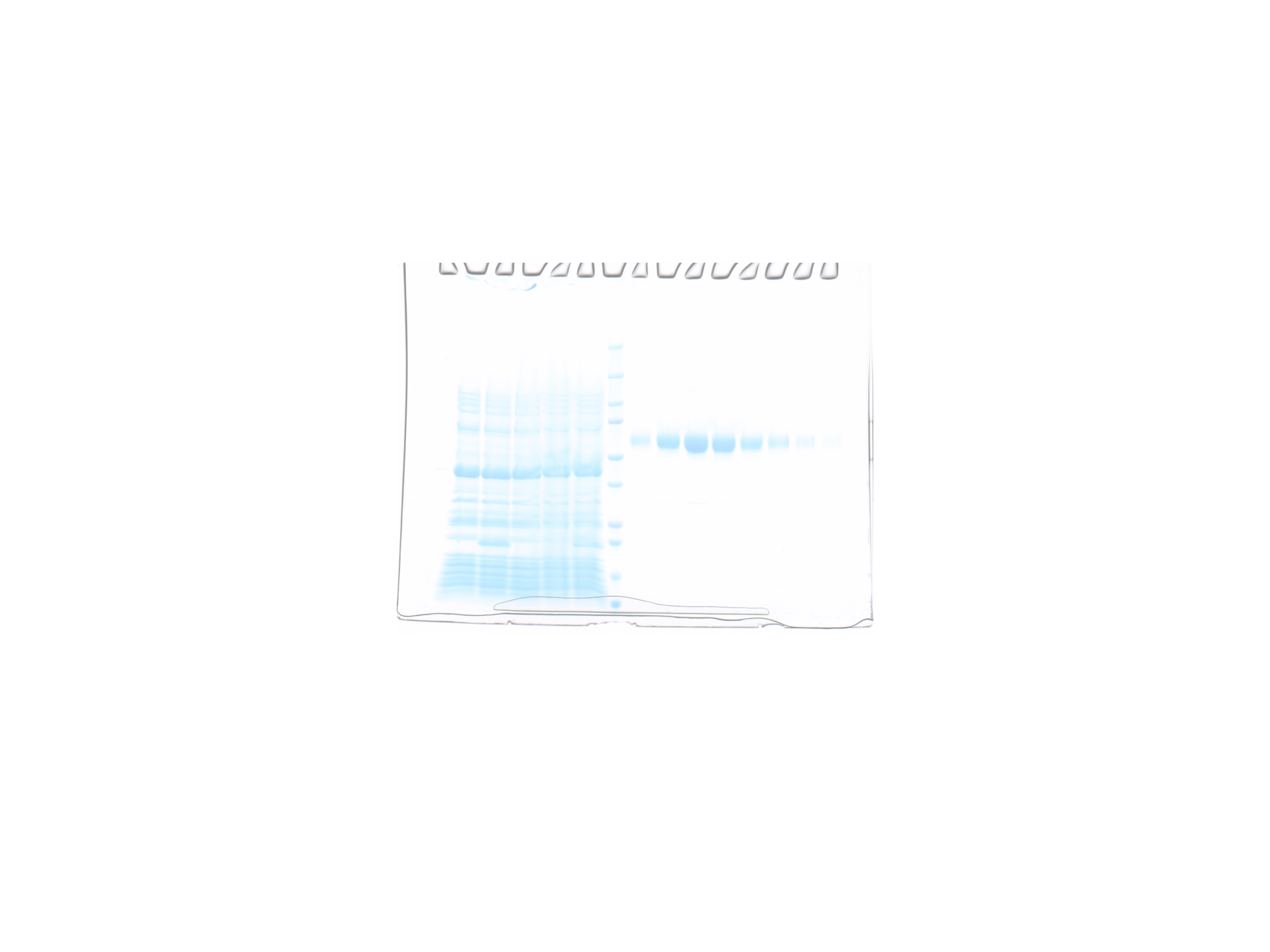

Supplement: Figure 3—source data 1. [file elife-99281-fig3-data1.zip › Figure 3-source data 1.tiff]

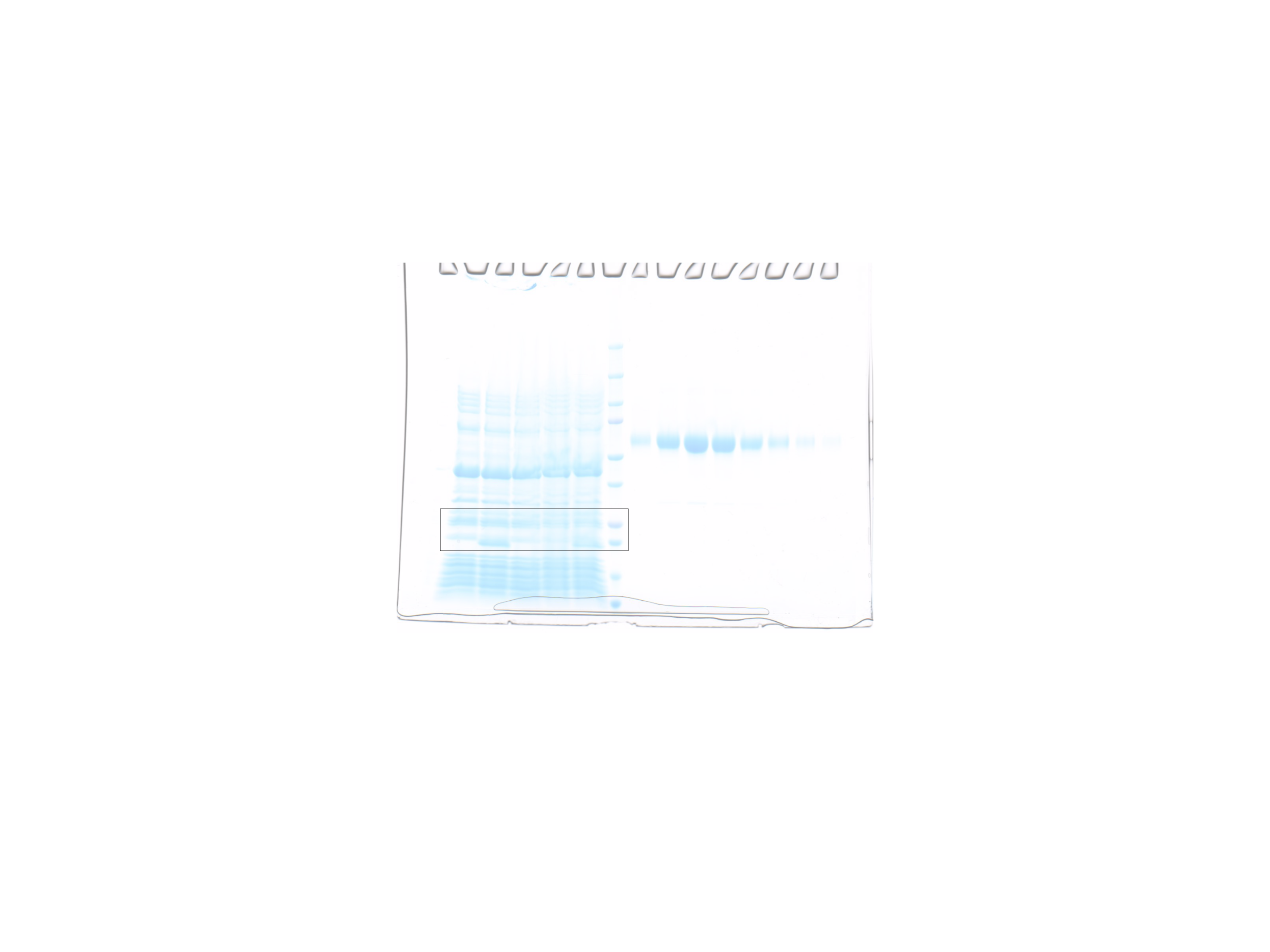

Supplement: Figure 3—source data 2. [file elife-99281-fig3-data2.zip › Figure 3-source data 1_labeled.tiff]
