# Supplementary material for: Oxydifficidin, a potent Neisseria gonorrhoeae antibiotic due to DedA-assisted uptake and ribosomal protein RplL sensitivity
Source: eLife. 2025 May 28;13:RP99281. doi: 10.7554/eLife.99281 (PMC12119084; doi:10.7554/eLife.99281)
Supplement: Figure 3—source data 4. [file elife-99281-fig3-data4.zip › Figure 3-source data 2_Labeled.pdf]

Assay Class: Eukaryote Total RNA Nano
Data Path: C:\... expert\data\Eukaryote Total RNA Nano_2023-09-29_Jingbo.xad

Created: 9/29/2023 2:51:54 PM
Modified: 9/29/2023 3:16:32 PM

**Gel Image**

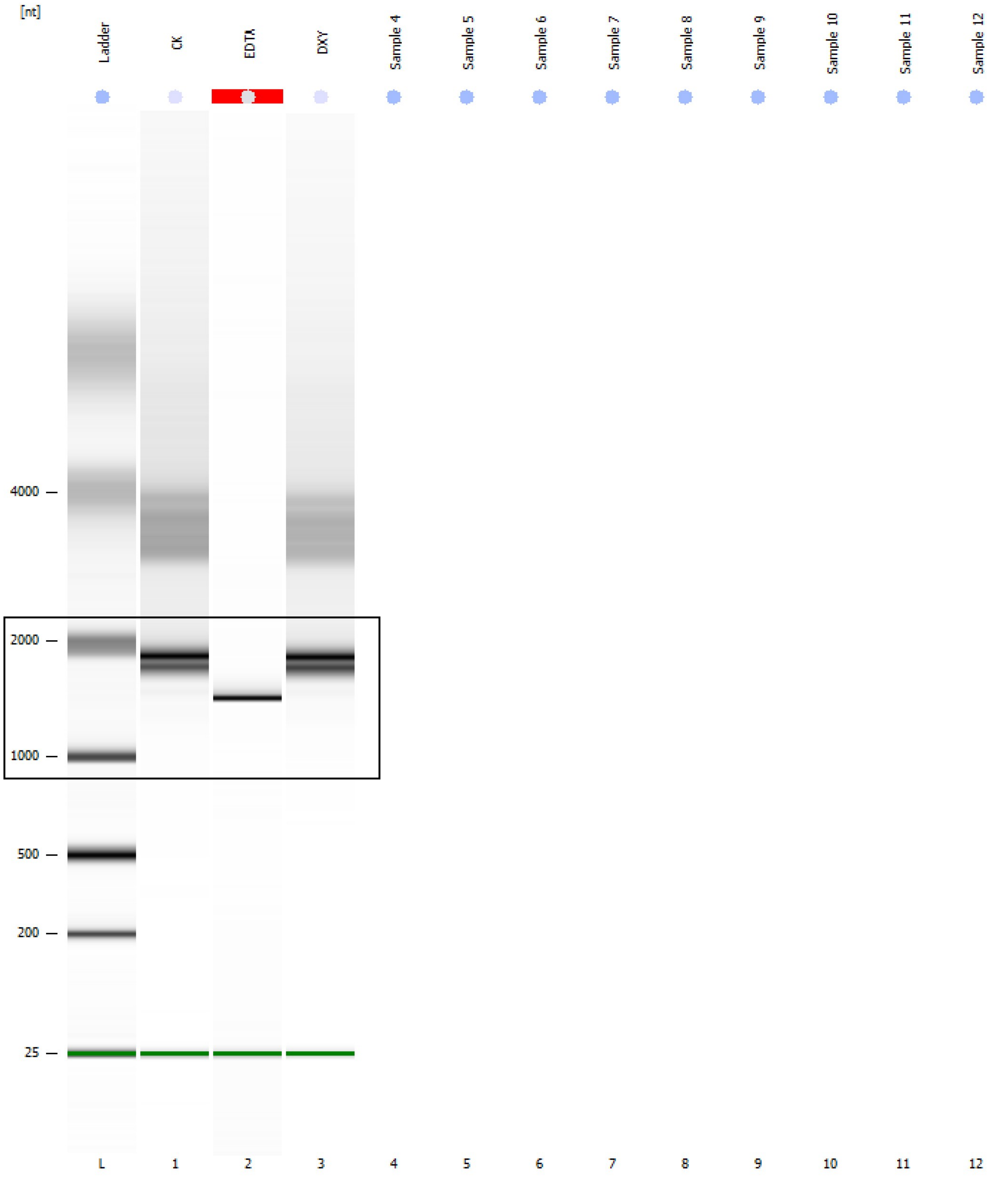


 © Copyright 2003 - 2009 Agilent Technologies, Inc.
